# An Examination of Hate-Motivated Behavior Among Adults in Scotland and Associations with Risk Factors for Self-Directed Violence

**DOI:** 10.1177/08862605241279393

**Published:** 2024-09-20

**Authors:** Kirsten Russell, Simon C. Hunter, Susan Rasmussen, Aideen Quirke, Robert J. Cramer

**Affiliations:** 1 University of Strathclyde, UK; 2 Glasgow Caledonian University, UK; 3 University of Western Australia; 4 University of North Carolina at Charlotte, USA

**Keywords:** hate crimes, measurement, microaggressions, defeat, entrapment

## Abstract

Hate-motivated behavior (HMB) ranges from microaggressions to criminal acts and is a public health concern with consequences for the physical and mental well-being of individuals, families, and communities. The Hate-Motivated Behavior Checklist (HMBC) was developed with the goal of advancing the measurement of HMB perpetration. To provide insights into perpetration and victimization across the HMB continuum in Scotland, the present study sought to examine the factor structure of both the original HMBC and our adapted victimization version in a sample of adults currently living in Scotland. It also aimed to test associations between HMB and cognitions, which are related to self-directed violence (defeat and entrapment). Participants (*n* = 447) completed an online cross-sectional survey assessing demographic factors, HMB (perpetration and victimization), and perceptions of defeat and entrapment. Confirmatory factor analysis was used to examine the factor structure of the HMBC and the adapted victimization version of this checklist and path analyses were implemented to provide insights into potential links between HMB, defeat, and entrapment. In line with previous work, results provided support for interpreting the HMB Checklist as a single-factor total score. This was also true for the victimization version of the checklist. Results indicated that HMB victimization (but not perpetration) was associated with increased perceptions of defeat and entrapment. These findings suggest that the HMBC (for assessing both perpetration and victimization) represents potentially useful tools for HMB research and supports their applicability outside of an American context. Furthermore, by examining HMB through the lens of a contemporary model of suicidal behavior, our findings also provide insights into potential psychological mechanisms linking interpersonal and self-directed violence. Future research should implement prospective research designs and integrate measures of self-directed violence outcomes alongside HMB, defeat, and entrapment, to further advance understanding of this association.

Hate-motivated behaviors (HMBs) represent a significant threat to public health. These behaviors can take many forms and exist on a continuum ranging from hate crimes or incidents to microaggressions (i.e., other behaviors that fall short of an illegal act) and are discriminatory in nature (Cramer et al., 2021). Indeed, HMBs are likely to involve repeat victimization (Home Office, 2018), and are reported to have a wide-ranging impact on the mental and physical well-being of its victims (Cramer et al., 2020). At a societal level, they can be socially divisive and can heighten tensions between communities (Hall, 2013). Despite the pernicious individual and societal effects, measurement of HMB perpetration and victimization is rather fragmented to date, with most measures focusing on either binary (e.g., Herek et al., 1999) experiences or focusing on HMB limited to one demographic/identity like gender (see, for example, Gartner et al., 2020).

The present study fills a measurement gap in the literature by examining the extent to which an established HMB perpetration measure, the Hate-Motivated Behavior Checklist (HMBC; Cramer et al., 2021, 2023), is a useful measure of HMB perpetration beyond the U.S. As this measure was developed and, to date, has been solely tested in America, the factor structure and applicability of the HMBC were examined within a Scottish sample. The present study also fills a measurement gap in the literature by translating the original HMBC to a victimization form and examining the factor structure of this adapted version of the checklist. This translation process involved adapting the original measure to ask participants whether they had experienced the same list of HMB but from the perspective of a victim (rather than a perpetrator). This work adds to the existing literature by capturing HMB victimization across the full spectrum of noncriminal to criminal HMBs.

Given that increasing evidence suggests that self-directed violence and interpersonal violence are linked and co-occur (Castellví et al., 2017), the present study also sought to examine links between HMB and self-directed violence-related cognitions with the goal of better understanding the psychological processes underpinning this association.

## Hate-Motivated Behavior in Scotland

In 2021 to 2022, Police Scotland recorded 6,927 hate crimes (Scottish Government, 2023). Since 2014 to 2015 the number of hate crimes has fluctuated between 6,300 and 7,000. In 2021 to 2022, just over three-fifths (62%) of hate crimes included a race aggravator, while over a quarter (27%) involved victimization due to sexual orientation. Other aggravators included disability (8%), religion (7%), and transgender identity (3%). Hate crimes may be motivated by more than one of the victim’s characteristics. In 2021 to 2022, 5% of these crimes had more than one recorded aggravator. When examining the nature of these hate crimes, the majority (53%) were recorded as threatening or abusive behavior, followed by racially aggravated conduct (13%) and common assault (13%). These statistics make it clear that HMB is a pressing problem in Scotland requiring further empirical attention.

The Scottish Crime and Justice Survey is a large-scale social survey that aims to provide a valid and reliable measure of adults’ experience of crime and complement police-recorded crime statistics. The survey does not ask directly about hate crime but does ask respondents who have experienced a violent crime to indicate whether they believe that any particular characteristic—perceived or actual—that they hold may have motivated the perpetrator. Over 40% of respondents thought that their most recent (or only) experience of harassment in the last year had been motivated by at least one of these characteristics (i.e., ethnicity, religion, sectarianism, gender identity, sexual orientation, disability, and age). It is important, however, to acknowledge that these figures likely do not reflect the true extent and nature of HMB within the country as these behaviors are often underreported by victims (Pezzella et al., 2019). In addition, although hate-motivated acts range from noncriminal/microaggressive behaviors (e.g., jokes and behavioral avoidance/shunning) to criminal and violent incidents (e.g., cyberstalking and physical assault) (Cramer et al., 2021), national statistics do not capture information regarding hateful acts that do not reach the criminal threshold (e.g., microaggressions). Moreover, current data sources lack the perspective of those who perpetrate these hateful acts. Capturing insights into the full range of HMB from the perspective of perpetrators is a key component of understanding the extent, nature, and drivers, of HMB and the development and evaluation of prevention and intervention efforts.

Research has demonstrated that the impact of HMB is far-reaching and that victims of HMB report more psychological distress and physical health difficulties compared to victims of non-hate crimes (e.g., Corcoran et al., 2015; Iganski & Lagou, 2015). The detrimental impact of microaggressions and hate crimes is suggested to be cumulative (Williams, 2019) and individuals who share similar characteristics to the victim can experience vicarious emotional trauma (Perry & Alvi, 2012; Walters et al., 2020). HMB can indicate to entire communities that they are not welcome or tolerated and, as a result, alters their perceptions of safety (Paterson et al., 2018). Given the damaging implications of these behaviors for both victims and communities, internationally, there is a need for researchers to implement measures that capture data across the continuum of noncriminal and criminal HMB and there is a need for measures to do so from the perspective of both victims and perpetrators.

## Development of the HMBC

Prior to the development of the HMBC, measurement of hate-motivated acts and their underlying drivers was piecemeal and instruments assessing HMB from a perpetrator perspective were lacking. The few exceptions included measures such as the self-report Mental Illness Microaggression Scale-Perpetrator (MIMS-P; Gonzales et al., 2015) and clinician-rated Bias Motivation Profile (Dunbar, 2003). Even these examples of perpetration measures fail to integrate the full range of HMB and do not capture underlying motivations for such behavior.

Partly due to the multitude of disciplines examining HMB, there is little integration of motivations driving hate-motivated acts within measurement tools. Motivations associated with the perpetration of hateful acts include but are not limited to peer influence, holding prejudiced attitudes, thrill-seeking, boredom, perceived defense of one’s ingroup against outgroups, retaliation, desire to be (in)famous, desire to exterminate outgroups, impulsivity, alcohol use, and poor social skills (e.g., Franklin, 2000; McDevitt et al., 2002; Parrott, 2008; Walters, 2011). A gap existed in the literature in pulling together these varied motivations in one assessment tool.

The HMBC (Cramer et al., 2021, 2023) is a three-part self-report instrument designed to assess: (a) the full spectrum of noncriminal to criminal HMBs (section I); (b) the extent to which behaviors target minoritized groups; and (c) the degree to which certain motivations play a part in one’s HMB. Section I (lifetime behaviors), rated in a discrete no/yes format, comprises a 26-item single-factor total score with high reliability (Cramer et al., 2023). Sections II (target groups) and III (motivations) are each rated on five-point scales and are typically used at the item level to contextualize respondents’ lifetime behavior scores. The HMBC behaviors total score demonstrates convergent validity with male gender, anti-sexual minority prejudice, and positive views of hate groups (Cramer et al., 2021). Perceived intrusion is consistently the most cited reason for the commission of HMB. HMB targeting persons based on race, ethnicity, sexual orientation, sex, and political affiliation are the most commonly cited characteristics (Cramer et al., 2021, 2023).

The HMBC provides a starting point for the study of hate-motivated acts in the United Kingdom. The measure was developed and, to date, has been tested solely in America. Given that there may be variability in the extent and nature of HMB cross-culturally, an important and useful next step in advancing HMB research is to test the applicability of this measure outside an American context. The Scottish Government has recently highlighted that tackling HMB is a key strategic priority within their national violence prevention framework and recognizes that robust data on the nature and extent of HMB is essential to more effectively preventing these behaviors in Scotland. Capturing insights from a perpetrator’s perspective is a key piece of this puzzle and represents an important component in informing appropriate interventions and policy development. As such, Scotland provides relevant context for testing the applicability of this measure.

## Adapting the HMB Checklist to Capture Insights into Victimization

Previous research has provided valuable insights into experiences of hate crime victimization internationally (e.g., see FRA, 2012, 2018). However, despite hate-motivated acts range from noncriminal/microaggressive behaviors to criminal and violent incidents (e.g., cyberstalking and physical assault) (Cramer et al., 2021), microaggressions (e.g., Fisher et al., 2019) are frequently measured separately from HMBs (e.g., Vergani et al., 2022), which limits the ability to capture the whole range of HMBs in an efficient manner. Even where there are measures of intersectional microaggressions, instruments often suffer a number of limitations such as not capturing the integrated experience of a person with intersecting identities (Singh et al., 2021).

HMB is common in Scotland. Yet it is likely that current figures do not reflect the true extent and nature of HMB within the country. Adapting the HMBC behaviors scale to capture lifetime victimization has the potential to provide a uniform way of measuring or quantifying this public health problem. Examining whether findings regarding the utility of the original HMBC behaviors scale are replicated in a non-American sample is a necessary step prior to undertaking and testing this adaptation. Moreover, the use of HMBC commission and victimization behavior scales in the same study would facilitate simultaneous consideration as related to novel application of self-directed violence, namely the Integrated Motivational-Volitional Model (IMV; O’Connor, 2011), to the study of HMB. We outline potential intersections between HMB victimization and a self-directed violence model in the following sections to establish the groundwork for the integrated study of hate-motivated perpetration, victimization, and cognitions associated with self-directed violence.

## The Potential Intersection of HMB and Self-Directed Violence

Suicide is a leading cause of death globally and it is estimated that for every person who has died by suicide, approximately 20 people have attempted to take their own life (WHO, 2014, 2019). Suicide-related phenomena comprise suicidal thoughts, suicide attempts, and death by suicide. Suicide-related behavior and interpersonal violence are typically treated as separate constructs and may initially seem distinct (Shafti et al., 2021). However, evidence supports the conceptualization of suicide-related behavior as self-directed violence (Cramer et al., 2017; WHO, 2014) and there is increasing evidence that self-directed violence and interpersonal violence are linked and co-occur (Castellví et al., 2017; O’Donnell et al., 2015; Slade, 2018; Stack, 2014).

To advance understanding regarding the joint conceptualization of self-directed and interpersonal violence, and inform both assessment and intervention efforts, Cramer et al. (2017) recommended that researchers: (a) examine additional risk factors; (b) employ behavioral checklists to measure perpetration; and (c) explore different subtypes of interpersonal violence. As limited research has focused on the role of psychological factors at the nexus of violent victimization, violent perpetration, and self-directed violence, we advance the existing literature by applying key psychological constructs from a leading model of self-directed violence (i.e., the Integrated Motivational-Volitional Model of Suicidal Behavior, IMV: O’Connor, 2011; O’Connor & Kirtley, 2018) to the study of HMB.

The IMV is a contemporary model of suicidal behavior (O’Connor, 2011). The three-phase model aims to provide a framework to aid understanding of how suicidal thoughts emerge and the factors that increase the likelihood that these thoughts will be translated into a suicide attempt. There is increasing evidence for the pathways and processes detailed within the IMV model (Branley-Bell et al., 2019; Dhingra et al., 2015; Russell et al., 2020). Central to the model is the assertion that increased feelings of defeat and entrapment underpin the emergence of the intention to harm oneself (Gilbert & Allan, 1998, O’Connor, 2011; O’Connor & Kirtley, 2018). More specifically, the model hypothesizes that when an individual perceives themselves to be trapped by internal (internal entrapment) and/or external factors (external entrapment) in their life, that they have a strong desire to escape from, they are more likely to experience thoughts of suicide. This intention to harm oneself emerges because engaging in suicidal behavior is seen as the salient solution to escaping thoughts, feelings, and/or life circumstances. Feelings of entrapment are thought to be triggered by perceptions of defeat/humiliation. The defeat-entrapment-suicide pathway is driven by a range of background vulnerability factors and stressful life events. Given that HMBs are linked with suicide-related outcomes (e.g., Duncan & Hatzenbuehler, 2014; Hollingsworth et al., 2017), it is plausible that experiences of interpersonal violence victimization or perpetration may serve such roles in driving feelings of entrapment and defeat. For example, experiencing HMB can have consequences for both physical and psychological functioning. This experience (and the associated impacts) may contribute to increased feelings of defeat/humiliation, which may in turn result in these individuals feeling trapped by their thoughts and feelings and/or life circumstances. Applying empirically supported IMV constructs (i.e., defeat and entrapment) to both HMB perpetration and victimization will advance our understanding of the psychological processes that underpin the intersection of these phenomena.

## The Current Study

The aims of the current study were twofold. First, to examine the factor structure of both the original and adapted victimization versions of the HMBC (HMBC-V) behaviors scale among adults in Scotland. The second intention was to test the associations between HMB (perpetration and victimization) and self-directed violence-related cognitions of defeat and entrapment. We had the following hypothesis (H):

H1: There will be a single-factor HMBC and HMBC-V behaviors scale (i.e., both HMBC and HMBC-V will be best interpreted as a single total score).
H2: HMB victimization and perpetration will be associated with defeat and entrapment.

## Methods

### Participants

Participants were recruited across several channels. The study was advertised on online platforms including Twitter and Facebook, and an advert was also placed on a virtual university research recruitment platform. Posters advertising the research were also placed around the campus of one university in Scotland. Table 1 contains sample demographic information. The following were predominant sample characteristics: young adult age (average almost 23 years old), low income, female gender (79.0%), Scottish national identity (83.2%), and White Scottish race/ethnicity (79.4%). Sexual orientation was quite diverse, with just over two-thirds of participants identifying as heterosexual and about one-third indicating a range of sexual minority identities (e.g., lesbian, pansexual, and asexual). Political affiliation varied considerably, with the most common affiliations being the Scottish National Party (43.4%), Labor (21.5%), and Green Party (12.8%). Religion was also diverse, with the following three most commonly indicated identities: Atheist (27.7%), Catholic (21.9%), and agnostic (13.6%). A variety of other religious identities (e.g., Muslim and Protestant) were also represented in the sample.

**Table 1.** Sample Demographic Information.

Variable	*n* (%)	*M* (*SD*)
Age	—	22.84 (7.27)
Estimated annual income	—	£13,346.98 (£12,620.58)
Gender		
Male	76 (17.0)	—
Female	353 (79.0)	—
Nonbinary	9 (2.0)	—
Transgender female	4 (0.9)	—
Transgender male	2 (0.4)	—
Queer	3 (0.7)	—
National identity		
Scottish	372 (83.2)	—
English	33 (7.4)	—
Northern Irish	8 (1.8)	—
British	3 (0.7)	—
Malaysian	5 (1.1)	—
Irish	4 (0.9)	—
Omani	3 (0.7)	—
Other	18 (4.0)	—
Missing	1 (0.2)	—
Ethnicity/race		
White Scottish	355 (79.4)	—
White Irish	8 (1.8)	—
White other British	29 (6.5)	—
White Polish	2 (0.4)	—
Asian/Asian Scottish/Asian British	21 (4.7)	—
African/African Scottish/African British	2 (0.4)	—
Biracial	3 (0.7)	—
Multiracial	7 (1.6)	—
Other White (e.g., Austrian)	12 (2.7)	—
Other	6 (1.3)	—
Missing	2 (0.4)	—
Sexual orientation		
Gay	10 (2.2)	—
Lesbian	16 (3.6)	—
Heterosexual	300 (67.1)	—
Bisexual	64 (14.3)	—
Queer	15 (3.4)	—
Questioning	6 (1.3)	—
Pansexual	13 (2.9)	—
Asexual	3 (0.7)	—
Prefer no label	19 (4.3)	—
Missing	1 (0.2)	—
Political affiliation		
Scottish national party	194 (43.4)	—
Conservative	10 (2.2)	—
Labor	96 (21.5)	—
Liberal democrats	16 (3.6)	—
Green party	57 (12.8)	—
Other (e.g., none)	56 (12.5)	—
Missing	18 (4.0)	—
Religion		
Catholic	98 (21.9)	—
Protestant	49 (11.0)	—
Baptist	1 (0.2)	—
Christian other	20 (4.5)	—
Muslim	10 (2.2)	—
Buddhist	3 (0.7)	—
Atheist	124 (27.7)	—
Agnostic	61 (13.6)	—
Spiritual	24 (5.4)	—
Other (e.g., no religion)	17 (3.6)	—
Prefer not to say	33 (7.4)	—
Missing	7 (1.6)	—

*Note.* *N* = 447. M = Mean; SD = Standard deviation.

### Measures

#### Demographics

Participants were asked to provide information regarding their age, gender identity, ethnicity, sexual orientation, personal financial status, political affiliations, and religious beliefs.

#### Hate-Motivated Behavior

History of engaging in HMBs was captured using the HMBC (Cramer et al., 2021). All three sections of the checklist were administered. Section I asks participants “How often have you engaged in the following actions (in your lifetime) toward or about another person based on knowing or believing you knew his or her demographic characteristic.” This section features 26 behavioral items that encompass violence (e.g., hit/punched a person), property crime (e.g., wrote graffiti), and noncriminal microaggressive acts (e.g., told jokes). Section II asks participants to indicate the extent to which they engaged in hateful acts because of specific perceived characteristics of the victim. There are 11 items, each relating to potential characteristics, including ethnicity, gender identity, sexual orientation, and national origin. Section III includes 15 items, each of which asks participants to report the extent to which their behaviors have been driven by a specific motivation including boredom, feeling threatened, revenge, and protecting their neighborhood. Internal consistency was shown to be excellent within the current sample (α = .88).

With the aim of assessing participants’ lifetime experiences of self-reported hate-motivated behavior victimization in Scotland, we adapted the first section (behavior scale) of the HMBC. The adapted checklist replicates the 26 behavioral items from the original measure but instead asks from the perspective of whether the participant has experienced the hateful acts themselves as a result of their own demographic characteristics (see Supplemental material for the full measure). Internal consistency was shown to be excellent within the current sample (α = .92). The decision to adapt only the first section (i.e., the behavior scale) of the HMBC for the purpose of assessing HMB victimization was guided by the assumption that while victims would be able to report accurately on the type of HMB that they had experienced, they would not always have a robust or tangible understanding as to why they had been the target of this behavior or of the perpetrators’ motivation for engaging in these hateful acts toward them.

#### Defeat

The Defeat Scale (Gilbert & Allan, 1998) is a 16-item measure that assesses an individual’s feelings of defeat (i.e., perceived failed struggle and loss of social rank). Respondents indicate on a five-point scale (ranging from 0 to 4) the occurrence of these perceptions. Scores for each item are summed to create a total continuous score with higher scores indicating greater levels of defeat. The measure has been widely used and has demonstrated concurrent validity with other measures of social rank (Griffiths et al., 2014). In the study, the measure displayed good/excellent internal consistency (α = .90).

#### Entrapment

Perceptions of being trapped were assessed using the 16-item Entrapment Scale (Gilbert & Allan, 1998). The six-item internal entrapment subscale reflects perceptions of entrapment by one’s own thoughts and feelings (e.g., “I feel trapped inside myself”). The 10-item external entrapment subscale reflects perceptions of entrapment by external situations (e.g., “I feel trapped by other people”). Respondents rate the extent to which each item describes their feelings on a five-point scale that ranges from 0 to 4. Responses to items are summed for each subscale to create a total score for both internal entrapment and external entrapment. Higher scores indicate greater levels of entrapment. The scale has been used extensively and both the internal entrapment (α = .91) and external entrapment (α = .90) subscales had excellent internal consistency in the current study.

### Procedure

This investigation adhered to the British Psychological Society’s ethical guidelines for internet-mediated research (BPS, 2021), and approval was obtained from the University Ethics Committee prior to commencing data collection. The investigation was conducted online via Qualtrics. As participants completed the online survey at one time point, the investigation was cross-sectional in nature. Data collection took place over a 5-month period (December 2022–April 2023). All individuals were informed that the project recruited anyone over the age of 18 living in Scotland and that the researchers were interested in experiences of violence from people living in Scotland. Participants were given access to a detailed information sheet, outlining the nature of the study, the contact details of the researchers, and information regarding relevant support organizations. Informed consent was requested through a tick box before participants were able to access the survey. After providing consent, participants were asked to complete a basic demographics questionnaire, followed by a range of measures which were presented in a randomized order. Participants were recruited as part of a larger HMB survey and were all invited to complete both the HMBC and HMBC-V. A range of other variables were measured but are not the central focus of this paper. Those relevant to the current paper are in *Measures* below. The survey took approximately 30 min on average to complete. Once participants had completed the survey, they were provided with a downloadable debrief sheet that restated the purpose of the study, provided contact details for researchers, and highlighted local mental health and victim support organizations. Participants who were recruited through the university research recruitment platform received course credits for taking part. No other payments or incentives for participation were provided.

### Data Analysis

Details relating to data preparation and cleaning are presented in the Supplemental material.

To test H1, Confirmatory Factor Analyses (CFA) were implemented using Mplus Version 7.31 (Los Angeles, CA, USA; Muthén & Muthén, 2008). CFA is used to confirm whether the data fits a hypothesized measurement model based on theory or prior research. As previous research (Cramer et al, 2021) indicated that HMB behaviors (as assessed by the HMBC) were best interpreted as a single total score, the first CFA evaluated the fit of a single-factor structure for the HMBC using all 26 items. The Weighted Least Squared Mean and Variance (WLSMV) adjusted estimator was used to account for the binary nature of the indicators. The second CFA repeated this procedure, testing a hypothesized one-factor model, but applied it to the HMBC-V and its associated items. The following indices of fit were used to evaluate each CFA: (a) The Root Mean Square Error of Approximation (RMSEA) and its 90% Confidence Interval, where a value of less than 0.05 is viewed as “good,” up to 1.00 is “mediocre,” and above 1.00 is “poor” (Byrne, 2016; Geiser, 2013); (b) the Comparative Fit Index (CFI) and Tucker-Lewis Index (TLI), where scores over 0.90 are considered “good” and those over 0.95 are considered “excellent” (Byrne, 2016; Geiser, 2013); (c) the Weighted Root Mean Square Residual (WRMR) where a score below 1.0 can be taken to reflect a good fitting model (Yu, 2002, cited in DiStefano et al., 2017).

To examine H2, a path analysis was estimated. Path analysis is used to evaluate the relationships between study variables and tested our hypotheses about the links between HMB (perpetration and victimization) and feelings of defeat and entrapment. In this case, the path analysis was estimated using scale scores as observed variables, with defeat, internal entrapment, and external entrapment regressed onto the HMBC-V and HMBC. This model used continuous scores and so the Maximum Likelihood with Robust Standard Errors (MLR) estimator was implemented in Mplus and this utilized Full Implementation Maximum Likelihood to address missing data. Both outcome variables were permitted to covary as were the three predictor variables. No fit indices were reported for the path analysis since it was a saturated model. Model syntax is presented in the Supplemental material.

## Results

### Confirmatory Factor Analyses

To examine the factor structure of the original HMBC (and determine how the checklist is best scored and interpreted), a CFA was implemented. Fit for the CFA model for the HMBC-V was, overall, satisfactory. The RMSEA was 0.080 (90% CI [0.075, 0.085]), CFI was 0.930, TLI was 0.924, and the WRMR was 1.934. All standardised coefficient (STDYX) estimates, akin to factor loadings, indicated that there were no poorly performing items (all estimates ≥0.559). To examine the factor structure of the HMBC-V, CFA was also implemented. Fit for the CFA model for the HMBC was also satisfactory. The RMSEA was 0.043 (90% CI [0.037, 0.049]), CFI was 0.947, TLI was 0.942, and the WRMR was 1.463. For the HMBC, the STDYX estimates indicated that there were no poorly performing items (all estimates ≥0.519). These findings support a single-factor HMBC and HMBC-V behaviors score, suggesting that HMBC behaviors (assessed from both a perpetrator and victim perspective) are best interpreted as a single total score (rather than separate subscales within each checklist).

### Descriptive Statistics

Table 2 displays correlations, means, standard deviations, and ranges for the five scale scores subsequently included in the path analysis. HMBC perpetration and victimization scores demonstrated a moderate positive correlation with one another. The HMBC-V behaviors score showed consistently significant small and positive associations with IMV outcomes. The HMBC perpetration behaviors score displays significant positive, but minute, correlations with IMV outcomes.

**Table 2.** Descriptive Statistics for, and Bivariate Correlations Between, Key Study Variable.

Variable	1	2	3	4	5
HMBC-V	—	.55***	.37***	.31***	.33***
HMBC		—	.16***	.11*	.15**
Defeat			—	.82***	.74***
External entrapment				—	.86***
Internal entrapment					—
Mean	8.25	3.91	23.32	9.87	4.53
Standard deviation	6.66	4.10	13.49	10.19	5.53
Range	0–26	0–26	0–64	0–40	0–24

*Note.* HMB = Hate-motivated behavior; HMBC = Hate-Motivated Behavior Checklist.

* Listwise deletion was implemented in SPSS28 (IBM Corp., Armonk, NY, USA), so *N* varied from 386 to 425.

Descriptive statistics and % of the sample endorsing HMBC targeted groups and motivations items are presented in Table 3 and the Supplemental material. When considering targeted groups, physical appearance, political affiliation, sex, sexual orientation, age, and ethnicity were the most endorsed by perpetrators. The most endorsed motivations were, others were doing it, perceived intrusion, and person made a threat.

**Table 3.** Hate-Motivated Behavior Targeted Groups and Motivations Sample Statistics.

HMBC Target Group	*M*	*SD*	% endorsed
1. Ethnicity	0.14	0.47	10.6
2. Gender identity	0.11	0.40	8.5
3. Sexual orientation	0.18	0.49	14.2
4. Religion	0.15	0.48	11.5
5. Disability	0.09	0.35	7.5
6. National origin	0.07	0.30	4.8
7. Sex	0.21	0.62	13.9
8. Age	0.17	0.51	12.6
9. Political affiliation	0.40	0.80	24.9
10. Physical appearance	0.42	0.76	29.8
HMBC Motivation	*M*	*SD*	% endorsed
1. You were bored	0.21	0.54	15.0
2. You wanted excitement	0.16	0.46	11.9
3. You wanted a thrill	0.14	0.45	10.2
4. The person(s) were intruding on you	0.64	0.93	39.2
5. The person threatened you	0.65	0.94	39.7
6. Of the person’s personal characteristic(s) only (e.g., race, and ethnicity)	0.07	0.29	5.8
7. You want to eliminate the world of the person’s group of people	0.02	0.18	1.8
8. The person and their group are taking away our resources (e.g., money, jobs, and food)	0.05	0.30	2.9
9. You were feeling impulsive	0.26	0.58	20.1
10. Others in my group were doing it	0.72	0.97	44.4
11. I was protecting my neighborhood from people like them	0.04	0.24	2.4
12. You wanted them to be in the news	0.02	0.23	1.2
13. You wanted to support a public figure who opposes this group	0.03	0.58	2.5
14. You wanted revenge because someone else from the person’s group did something negative	0.24	0.51	17.1

*Note.* Sample statistics for the Hate-Motivated Behavior Target Population and Motivation scales. *N* = 447. HMBC = Hate-Motivated Behavior Checklist; SD = Standard deviation.

### Path Analysis

**Figure 1.**
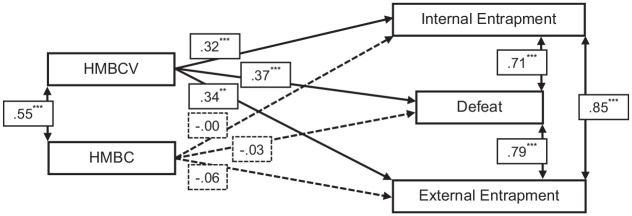
Path analysis results. NB. Solid lines indicate significant paths, dashed lines indicate nonsignificant paths.

To examine if HMB victimization and perpetration were associated with increased feelings of defeat and entrapment, path analysis was conducted. The HMBC-V and the HMBC were significantly, and positively correlated as were both entrapment scores, defeat with internal entrapment, and defeat with external entrapment (see Figure 1). The HMBC-V was significantly and positively associated with all three outcome measures while, at the same time, the HMBC was not significantly associated with any of the three outcome measures. The model accounted for 12.8% of the variance in defeat, 9.6% of the variance in internal entrapment, and 10.1% of the variance in external entrapment.

## Discussion

Comprehensive measurement of HMB victimization and perpetration is largely lacking in the literature. To address this matter, the current study examined the factor structure of both the original and an adapted victimization version of the HMBC in a sample of adults’ living in Scotland. It also tested associations between HMB (perpetration and victimization) and perceptions of defeat, internal entrapment, and external entrapment. We found robust support for a single-factor structure with high reliability for both the HMBC and HMBC-V behaviors score (suggesting that both measures are best interpreted as a single total score). HMB victimization, but not perpetration, was associated with all IMV-related outcomes in the path model.

Our findings supported a moderate association between HMB perpetration and victimization. While evidence from the field of criminology consistently supports the robust overlap between victimization and offending (Jennings et al., 2012), this finding is noteworthy as research examining the victim-offender relationship in the HMB context is scarce. Those involved in engaging in interpersonal violence are less likely to report their own experiences of victimization (Berg & Felson, 2018). As such, this group warrants further investigation, within an HMB context, to ensure that they receive the services and support that they need.

### Examining the Factor Structure of the HMBC and HMBC-V

The current study sought to build on existing work relating to HMB by conducting further psychometric investigation of the original HMBC (Cramer et al., 2021, 2023) and providing novel insights into the factor structure of the adapted victimization version of the checklist (HMBV-C). Results of the confirmatory factor analysis demonstrated that HMBC behaviors (as captured from the perspective of the perpetrator) were best interpreted as a single-factor total score. These findings support our hypothesis and are in line with previous research conducted within an American context (Cramer et al., 2021). The result provides support for use of the HMBC in the study of HMB perpetration in Scotland. For example, the HMBC may be applied in population-based studies of interpersonal violence, as well as future research investigating social science theories of crime perpetration in community settings.

The HMBC-V, which sought to quantify lifetime experiences of HMB victimization, was also best interpreted as a single-factor total score. Cross-cultural research highlights that, at an international level, progress toward a cumulative and comparative evidence base in relation to HMB is limited by variability in definitions, terminology, criteria, and disconnected data sources (Sheppard et al., 2021; Vergani et al., 2022). The HMBC was developed in pursuit of a singular measurement tool that could overcome these limitations. Specifically, we provide the first evidence for the HMBC-V and its predecessor outside of the United States, a needed first step toward advancing cross-national HMB research. The addition of the victimization behavior scale to this measure represents a first step toward the goal of deriving a uniform approach to quantifying both lifetime HMB perpetration and victimization. Also, these findings set up a number of next research steps. For instance, establishment of both the HMBC and HMBC-V provides the opportunity the examine the victim-offender overlap concerning hateful acts. Moreover, the HMBC and HMBC-V can be applied in other Westernized and non-western nations to continue building a cross-national understanding of HMB victimization and perpetration. Such research may necessitate translation of the measures.

### Evaluating Links Between HMB and Feelings of Defeat and Entrapment

The second aim of the present study was to examine the link between HMB experiences and perceptions of defeat and entrapment, which are robust and proximal predictors of self-directed violence (O’Connor, 2011; O’Connor & Kirtley, 2018). Empirical evidence supports an association between interpersonal violence and self-directed violence across student, community, clinical, and forensic populations (e.g., Cramer et al., 2017; Slade, 2018). Given the overlap of violence subtypes, we explored the possible role of HMB victimization and perpetration within the IMV (O’Connor & Kirtley, 2018), namely defeat, internal entrapment, and external entrapment. The victimization scale of HMBC was positively associated with perceptions of all IMV outcomes. These findings support previous work, which highlights that experiencing violent victimization (e.g., Hanson et al., 2023; McManus et al., 2022) and HMBs (e.g., Duncan & Hatzenbuehler, 2014; Hollingsworth et al., 2017) are associated with increased suicide-related behaviors. Our research extends current understanding regarding the intersection between interpersonal and self-directed violence by (a) providing insights into the impact of experiencing HMB specifically and (b) highlighting the relevance of defeat and entrapment (internal and external) within this context. Previous evidence emphasizes the role of defeat and entrapment as potentially transdiagnostic psychological constructs underlying pathways to suicidal and self-harming thoughts and behaviors (Griffiths et al., 2014; O’Connor et al., 2018; Owen et al., 2018; Russell et al., 2020). This novel contribution to the literature offers preliminary evidence that entrapment and defeat may be an explanatory connection or contributing factor to the shared experience of HMB victimization and suicide. Future scholarly inquiry can integrate self-directed violence outcomes with HMB and IMV cognitions to test this possibility.

Those who perpetrate HMB tend to belong to majority (dominant) groups within society (Chakraborti et al., 2014; Walter et al., 2016) who have greater social power relative to those who belong to minority groups. Experiencing these hateful acts at the hands of these individuals may therefore leave victims feeling defeated, humiliated, and trapped. Indeed, Minority Stress Theory (Meyer, 1995, 2003), which was developed with a focus on sexual and gender minority people, theorizes that greater social stresses faced by individuals from minority groups, such as minority identity-related stigma, discrimination, and victimization, leave them at higher risk for negative mental health outcomes. HMB is an example of this victimization and can be conceptualized as a stressor that is driven and motivated by an individual’s stigmatized identity. This process is likely to act as a risk factor for self-harm and suicidal ideation and future research should directly investigate this possibility.

Our findings also demonstrated no significant link between HMB perpetration and IMV cognitions. As this is the first study to examine this relationship, replication in samples across different communities and nations is recommended. In particular, as this data was gathered from a community-based sample; future research should aim to oversample those at greater risk of engaging in HMB (e.g., young adult men and justice-involved persons; Roberts et al., 2013).

### Implications

This investigation builds on previous research focusing on the measurement of HMB, by (a) implementing a comprehensive assessment tool to provide insights into both the perpetration and victimization of HMB in a Scottish context and (b) testing the original and adapted behavior scale factors structure via the application of CFA. The replication of the single-factor structure on the HMBC supports its applicability outside an American context. Future research that utilizes this measure will continue to advance understanding of the prevalence of HMB and the motivations that underpin these acts, internationally. From a victimization perspective, it is important to recognize that HMB is underreported and that police-recorded figures do not fully reflect the true extent and nature of HMB (Pezzella et al., 2019). As such, the adapted self-report victimization behavior scale is important from a public health surveillance perspective. Systematically collecting data on the characteristics and magnitude of these behaviors is a key component of the public health approach to violence prevention and to developing and evaluating interventions aimed at perpetrators and victims of HMB nationally and internationally. Approaches may include legal and law enforcement strategies, educational approaches, public health programming, and psychological (i.e., interpersonal and individual level) strategies (Cramer et al., 2020).

The results of the current study have important theoretical implications. This investigation sought to extend what is known about the intersection between interpersonal and self-directed violence by examining HMB through the lens of a contemporary model of suicidal behavior, the IMV (O’Connor, 2011; O’Connor & Kirtley, 2018). Our findings highlight that this framework is relevant to people who have experienced interpersonal violence, especially HMB. Victims of HMB victimization were associated with IMV-related cognitions, but perpetration was not. Given the central role of defeat and entrapment within the IMV our findings provide preliminary evidence that victimization via HMB could act as a stressor that is associated with greater feelings of defeat and entrapment.

### Limitations and Future Directions

The cross-sectional nature of the survey data means that it is not possible to draw conclusions about the temporal ordering of the variables of interest. Future research employing prospective designs will be key to providing important temporal information regarding the identified relationships between HMB, defeat, and entrapment and, therefore, understanding how the psychological impacts of HMB unfold over time. Second, our sample lacked diversity in terms of gender, age, ethnicity, and nationality. Such limitations may restrict capturing the full prevalence or scope of HMB victimization and perpetration and limit the generalizability of our findings. Research suggests that those who hold multiple minority identities experience greater discrimination than those holding a single minority identity, and therefore also report a greater negative impact (Boyle & McKinzie, 2021; Millar & Brooks, 2021). Our sample did not contain sufficient diversity to examine the effects of intersecting minority identities on HMB, defeat, and entrapment. Future research should seek to oversample groups who are known to be at greater risk for involvement in HMB.

## Supplemental Material

sj-docx-1-jiv-10.1177_08862605241279393 – Supplemental material for An Examination of Hate-Motivated Behavior Among Adults in Scotland and Associations with Risk Factors for Self-Directed Violence Supplemental material, sj-docx-1-jiv-10.1177_08862605241279393 for An Examination of Hate-Motivated Behavior Among Adults in Scotland and Associations with Risk Factors for Self-Directed Violence by Kirsten Russell, Simon C. Hunter, Susan Rasmussen, Aideen Quirke and Robert J. Cramer in Journal of Interpersonal Violence
