# Supplementary material for: An Examination of Hate-Motivated Behavior Among Adults in Scotland and Associations with Risk Factors for Self-Directed Violence
Source: J Interpers Violence. 2024 Sep 20;40(11-12):2784–808. doi: 10.1177/08862605241279393 (PMC12048733; doi:10.1177/08862605241279393)
Supplement: sj-docx-1-jiv-10.1177_08862605241279393 – Supplemental material for An Examination of Hate-Motivated Behavior Among Adults in Scotland and Associations with Risk Factors for Self-Directed Violence [file sj-docx-1-jiv-10.1177_08862605241279393.docx]

**Online Supplement**

**Supplement 1**

HMBC-V Section I: Behaviors

Instructions: Using the scale below, please tell us if you have experienced the following actions (in your lifetime) as a result of another person knowing or believing they know your demographic characteristics (i.e., race, ethnicity, gender, sexual orientation, religion, disability, or national origin). That is, we are interested in the extent to which you have experienced these behaviors from others because of your race, ethnicity, gender, sexual orientation, religion, disability, and so on.

0 = No 1 = Yes

No Yes

1. Slurs or belittling name-calling:

2. Jokes at your expense:

3. Verbally threatened:

4. Been followed:

5. Received repeated, unwanted emails:

6. Received repeated, unwanted text messages:

7. Received repeated, unwanted phone calls/voice messages:

8. Been spat at:

9. Had insults yelled at you (other than slurs or belittling name-calling):

10. Had objects thrown at you:

11. Had your property damaged (e.g., technology, mailbox, home):

12. Been hit/punched:

13. Been pushed/shoved:

14. Been hit with object (e.g., bat, stick):

15. Been physically fought with (e.g., prolonged encounter using fists, slapping, kicking, tripping):

16. Someone attempted to physically fight you:

17. Unwanted sexual contact (e.g., touching, grinding):

18. Forced sex:

19. Attempted forced sex:

20. Been stolen from:

21. Had graffiti targeted at you:

22. Been stared or scowled at:

23. Been the target of negative talk (e.g., rumors, gossip, defaming):

24. Received hostile posts on your person’s social media page (e.g., Facebook, Twitter):

25. Been forced to behave a certain way (i.e., bullying):

26. Had someone avoid interpersonal contact with you:

27. Other actions (Please specify the other behaviours that you have experienced): ______________________________________________________________________________________________________________________________________________________

**Supplement 2**

**Data preparation.** A total of 692 persons opened the survey link and consented to participate. We approached data cleaning in a multi-step process consistent with best practice literature in ensuring data quality in online or internet survey research (e.g., Aust et al., 2013; Leiner, 2019). First, we dropped respondents with less than 70% complete data or with complete missing data on > 2 measures of interest, resulting in 174 case deletions. Most of these deleted cases had no or very few item responses. Second, we dropped extreme cases with respect to time to completion (i.e., those lower than the 5th and higher than the 95th percentile) because brief or extensive time to completion can signal inattentive or other poor responding (Leiner, 2019). This step resulted in 54 case deletions. Finally, we adopted a stringent approach to preventing duplicate responding (Aust et al., 2013) by dropping cases of repetitive IP addresses, resulting in 16 additional case deletions. The final analyzable sample size was 447, a sample proportion consistent with simulation research on data cleaning best practices (Arevalo et al., 2022).

**Supplement 3**. Mplus syntax for confirmatory factor analysis of HMBCV

**TITLE**:

Paper 1 CFA of HMBCV

**DATA**:

FILE = StrathHMBCDataForMPlus Short.dat;

**VARIABLE**:

NAMES ARE HMBCV1 HMBCV2 HMBCV3 HMBCV4 HMBCV5 HMBCV6

HMBCV7 HMBCV8 HMBCV9 HMBCV10 HMBCV11 HMBCV12

HMBCV13 HMBCV14 HMBCV15 HMBCV16 HMBCV17

HMBCV18 HMBCV19 HMBCV20 HMBCV21 HMBCV22

HMBCV23 HMBCV24 HMBCV25 HMBCV26

HMBC1 HMBC2 HMBC3 HMBC4 HMBC5 HMBC6 HMBC7

HMBC8 HMBC9 HMBC10 HMBC11 HMBC12 HMBC13

HMBC14 HMBC15 HMBC16 HMBC17 HMBC18 HMBC19

HMBC20 HMBC21 HMBC22 HMBC23 HMBC24 HMBC25

HMBC26 HMBCVTot HMBCPTot DepTot AnxTot DefTot

ExtEnt IntEnt WellbTot VxP;

USEVARIABLES ARE HMBCV1 HMBCV2

HMBCV3 HMBCV4 HMBCV5 HMBCV6

HMBCV7 HMBCV8 HMBCV9 HMBCV10 HMBCV11 HMBCV12

HMBCV13 HMBCV14 HMBCV15 HMBCV16 HMBCV17

HMBCV18 HMBCV19 HMBCV20 HMBCV21 HMBCV22

HMBCV23 HMBCV24 HMBCV25 HMBCV26;

CATEGORICAL ARE HMBCV1 HMBCV2

HMBCV3 HMBCV4 HMBCV5 HMBCV6

HMBCV7 HMBCV8 HMBCV9 HMBCV10 HMBCV11 HMBCV12

HMBCV13 HMBCV14 HMBCV15 HMBCV16 HMBCV17

HMBCV18 HMBCV19 HMBCV20 HMBCV21 HMBCV22

HMBCV23 HMBCV24 HMBCV25 HMBCV26;

MISSING ARE ALL (999.00);

**ANALYSIS**:

TYPE=GENERAL;

ESTIMATOR = WLSMV;

**MODEL**:

VFactor BY HMBCV1 HMBCV2

HMBCV3 HMBCV4 HMBCV5 HMBCV6

HMBCV7 HMBCV8 HMBCV9 HMBCV10 HMBCV11 HMBCV12

HMBCV13 HMBCV14 HMBCV15 HMBCV16 HMBCV17

HMBCV18 HMBCV19 HMBCV20 HMBCV21 HMBCV22

HMBCV23 HMBCV24 HMBCV25 HMBCV26;

**OUTPUT**:

SAMPSTAT;

STANDARDIZED;

MODINDICES;

**Supplement 4**. Mplus syntax for confirmatory factor analysis of HMBC

**TITLE**:

Paper 1 CFA of HMBC

**DATA**:

FILE = StrathHMBCDataForMPlus Short.dat;

**VARIABLE**:

NAMES ARE HMBCV1 HMBCV2 HMBCV3 HMBCV4 HMBCV5 HMBCV6

HMBCV7 HMBCV8 HMBCV9 HMBCV10 HMBCV11 HMBCV12

HMBCV13 HMBCV14 HMBCV15 HMBCV16 HMBCV17

HMBCV18 HMBCV19 HMBCV20 HMBCV21 HMBCV22

HMBCV23 HMBCV24 HMBCV25 HMBCV26

HMBC1 HMBC2 HMBC3 HMBC4 HMBC5 HMBC6 HMBC7

HMBC8 HMBC9 HMBC10 HMBC11 HMBC12 HMBC13

HMBC14 HMBC15 HMBC16 HMBC17 HMBC18 HMBC19

HMBC20 HMBC21 HMBC22 HMBC23 HMBC24 HMBC25

HMBC26 HMBCVTot HMBCPTot DepTot AnxTot DefTot

ExtEnt IntEnt WellbTot VxP;

USEVARIABLES ARE HMBC1 HMBC2 HMBC3 HMBC4

HMBC5 HMBC6 HMBC7 HMBC8 HMBC9 HMBC10 HMBC11

HMBC12 HMBC13 HMBC14 HMBC15 HMBC16 HMBC17

HMBC18 HMBC19 HMBC20 HMBC21 HMBC22 HMBC23

HMBC24 HMBC25 HMBC26;

CATEGORICAL ARE HMBC1 HMBC2 HMBC3 HMBC4

HMBC5 HMBC6 HMBC7 HMBC8 HMBC9 HMBC10 HMBC11

HMBC12 HMBC13 HMBC14 HMBC15 HMBC16 HMBC17

HMBC18 HMBC19 HMBC20 HMBC21 HMBC22 HMBC23

HMBC24 HMBC25 HMBC26;

MISSING ARE ALL (999.00);

**ANALYSIS**:

TYPE=GENERAL;

ESTIMATOR = WLSMV;

**MODEL**:

PFactor BY HMBC1 HMBC2 HMBC3 HMBC4

HMBC5 HMBC6 HMBC7 HMBC8 HMBC9 HMBC10 HMBC11

HMBC12 HMBC13 HMBC14 HMBC15 HMBC16 HMBC17

HMBC18 HMBC19 HMBC20 HMBC21 HMBC22 HMBC23

HMBC24 HMBC25 HMBC26;

**OUTPUT**:

SAMPSTAT;

STANDARDIZED;

MODINDICES;

**Supplement 5.** Factor loadings from both Confirmatory Factor Analyses

| **Item** | **HMBC** | **HMBCV** |
| --- | --- | --- |
| 1. Slurs or belittling name-calling | 0.72 | 0.76 |
| 2. Jokes at your expense | 0.73 | 0.84 |
| 3. Verbally threatened | 0.83 | 0.82 |
| 4. Been followed | 0.74 | 0.66 |
| 5. Received repeated, unwanted emails | 0.78 | 0.69 |
| 6. Received repeated, unwanted text messages | 0.55 | 0.83 |
| 7. Received repeated, unwanted phone calls/voice messages | 0.62 | 0.84 |
| 8. Been spat at | 0.80 | 0.72 |
| 9. Had insults yelled at you (other than slurs or belittling name-calling) | 0.83 | 0.83 |
| 10. Had objects thrown at you | 0.82 | 0.79 |
| 11. Had your property damaged (e.g., technology, mailbox, home) | 0.88 | 0.79 |
| 12. Been hit/punched | 0.94 | 0.89 |
| 13. Been pushed/shoved | 0.96 | 0.92 |
| 14. Been hit with object (e.g., bat, stick) | 0.94 | 0.90 |
| 15. Been physically fought with (e.g., prolonged encounter using fists, slapping, kicking, tripping) | 0.80 | 0.60 |
| 16. Someone attempted to physically fight you | 0.52 | 0.66 |
| 17. Unwanted sexual contact (e.g., touching, grinding) | 0.53 | 0.66 |
| 18. Forced sex | 0.77 | 0.65 |
| 19. Attempted forced sex | 0.73 | 0.56 |
| 20. Been stolen from | 0.75 | 0.83 |
| 21. Had graffiti targeted at you | 0.77 | 0.81 |
| 22. Been stared or scowled at | 0.67 | 0.66 |
| 23. Been the target of negative talk (e.g., rumors, gossip, defaming) | 0.56 | 0.80 |
| 24. Received hostile posts on your person’s social media page (e.g., Facebook, Twitter) | 0.66 | 0.74 |
| 25. Been forced to behave a certain way (i.e., bullying) | 0.87 | 0.83 |
| 26. Had someone avoid interpersonal contact with you | 0.82 | 0.78 |

NB. Items given in table above at taken from the HMBCV. For the HMBC the equivalent perpetrator items apply.

**Supplement 6**. Mplus syntax for Path analysis

**TITLE**:

Paper 1 Path analysis reversed

**DATA**:

FILE = StrathHMBCDataForMPlus Short.dat;

**VARIABLE**:

NAMES ARE HMBCV1 HMBCV2 HMBCV3 HMBCV4 HMBCV5 HMBCV6

HMBCV7 HMBCV8 HMBCV9 HMBCV10 HMBCV11 HMBCV12

HMBCV13 HMBCV14 HMBCV15 HMBCV16 HMBCV17

HMBCV18 HMBCV19 HMBCV20 HMBCV21 HMBCV22

HMBCV23 HMBCV24 HMBCV25 HMBCV26

HMBC1 HMBC2 HMBC3 HMBC4 HMBC5 HMBC6 HMBC7

HMBC8 HMBC9 HMBC10 HMBC11 HMBC12 HMBC13

HMBC14 HMBC15 HMBC16 HMBC17 HMBC18 HMBC19

HMBC20 HMBC21 HMBC22 HMBC23 HMBC24 HMBC25

HMBC26 HMBCVTot HMBCPTot DepTot AnxTot DefTot

ExtEnt IntEnt WellbTot VxP;

USEVARIABLES ARE HMBCVTot HMBCPTot DefTot ExtEnt IntEnt;

MISSING ARE ALL (999.00);

**ANALYSIS**:

TYPE=GENERAL;

ESTIMATOR = MLR;

**MODEL**:

DefTot WITH ExtEnt IntEnt;

ExtEnt WITH IntEnt;

HMBCVTot WITH HMBCPTot;

DefTot on HMBCVTot HMBCPTot;

ExtEnt on HMBCVTot HMBCPTot;

IntEnt on HMBCVTot HMBCPTot;

ExtEnt IntEnt DefTot HMBCVTot HMBCPTot;

! Estimates variances to ensure all cases are used

**OUTPUT**:

SAMPSTAT;

STANDARDIZED;

MODINDICES;
